# Impact of Obesity on Surgical Outcomes in Patients Undergoing Emergency Laparotomy: A Prospective Observational Study

**DOI:** 10.7759/cureus.85887

**Published:** 2025-06-12

**Authors:** Sudhir Marahanumaiah, Nitish Suresh, Bhoomika Rajkumar, Anubhav Priyadarshi

**Affiliations:** 1 Department of General Surgery, Kempegowda Institute of Medical Sciences, Bangalore, IND

**Keywords:** body mass index, laparotomy, length of stay, obesity, operative time, post-operative complications, wound healing

## Abstract

Background and aim: Obesity significantly impacts the outcomes of patients undergoing emergency laparotomy. It complicates emergency laparotomy by prolonging operative times, increasing risks due to comorbidities, impairing wound healing, and heightening respiratory complications, leading to higher morbidity, mortality, and healthcare costs. This study aimed to evaluate the impact of obesity on post-operative outcomes in patients undergoing emergency laparotomy at Kempegowda Institute of Medical Sciences (KIMS) Hospital and Research Centre, Bangalore. It focused on the association between body mass index (BMI) and the incidence of post-operative complications, length of hospital stay, and operative time.

Materials and methods: A total of 72 emergency laparotomy patients were grouped by BMI, normal vs. obese, as per the World Health Organization (WHO) classification. Comorbidities and surgical procedures were recorded, excluding laparoscopic cases and underweight patients. Post-operative complications were classified (Clavien-Dindo) and a comprehensive complication index assessed morbidity/mortality during hospitalization.

Results: The study included patients aged 18-88 years, with 48 having normal BMI and 24 classified as obese. Post-operative complications occurred more frequently in obese patients with comorbidities - diabetes (58.3%), hypertension (50%), CKD (4.2%), and IHD (4.2%) - compared to those with normal BMI (diabetes: 14.6%, hypertension: 20%). Notably, 66.7% of normal BMI patients had no complications. Obese patients had significantly longer operative times (by 44.23 minutes) and hospital stays (by 3.5 days).

Conclusion: The study demonstrates a significant impact of obesity on post-operative outcomes in patients undergoing emergency laparotomy. Additionally, the data show that a majority of patients with a normal BMI (66.7%), regardless of whether they had comorbid conditions, did not encounter any complications.

## Introduction

Obesity, defined as having a body mass index (BMI) of 30 kg/m² or higher, is rapidly increasing in prevalence within modern healthcare settings [1]. In India, the prevalence of obesity has increased rapidly in the last decade. In 2017-2020, 23% of all adults in India were obese [2]. Due to the increased risk of morbidity and mortality, obesity is now being recognized as a disease in its own right. Additionally, obesity is strongly associated with other metabolic disorders, including diabetes, hypertension, dyslipidemia, cardiovascular disease, and even some cancers [3]. Individuals with obesity have higher rates of mortality and morbidity compared to non-obese individuals [4,5].

Although obesity is perceived as a potential risk factor for adverse post-surgical outcomes [6-8], reports across a wide variety of surgical studies are conflicting [9]; therefore, the effect of obesity on surgical outcomes is not entirely clear, which is known as the obesity paradox [10,11]. Emergency laparotomy/laparoscopy (EL) is occasionally performed in patients who are obese, and the surgical outcome can potentially be impacted by the weight burden [12]. Therefore, this study aimed to evaluate the impact of obesity on post-operative outcomes in patients undergoing emergency laparotomy in Kempegowda Institute of Medical Sciences (KIMS) Hospital and Research Centre, Bangalore, focusing on the association between body mass index (BMI) and the incidence of post-operative complications, length of hospital stay, and operative time.

## Materials and methods

**Figure 1:**
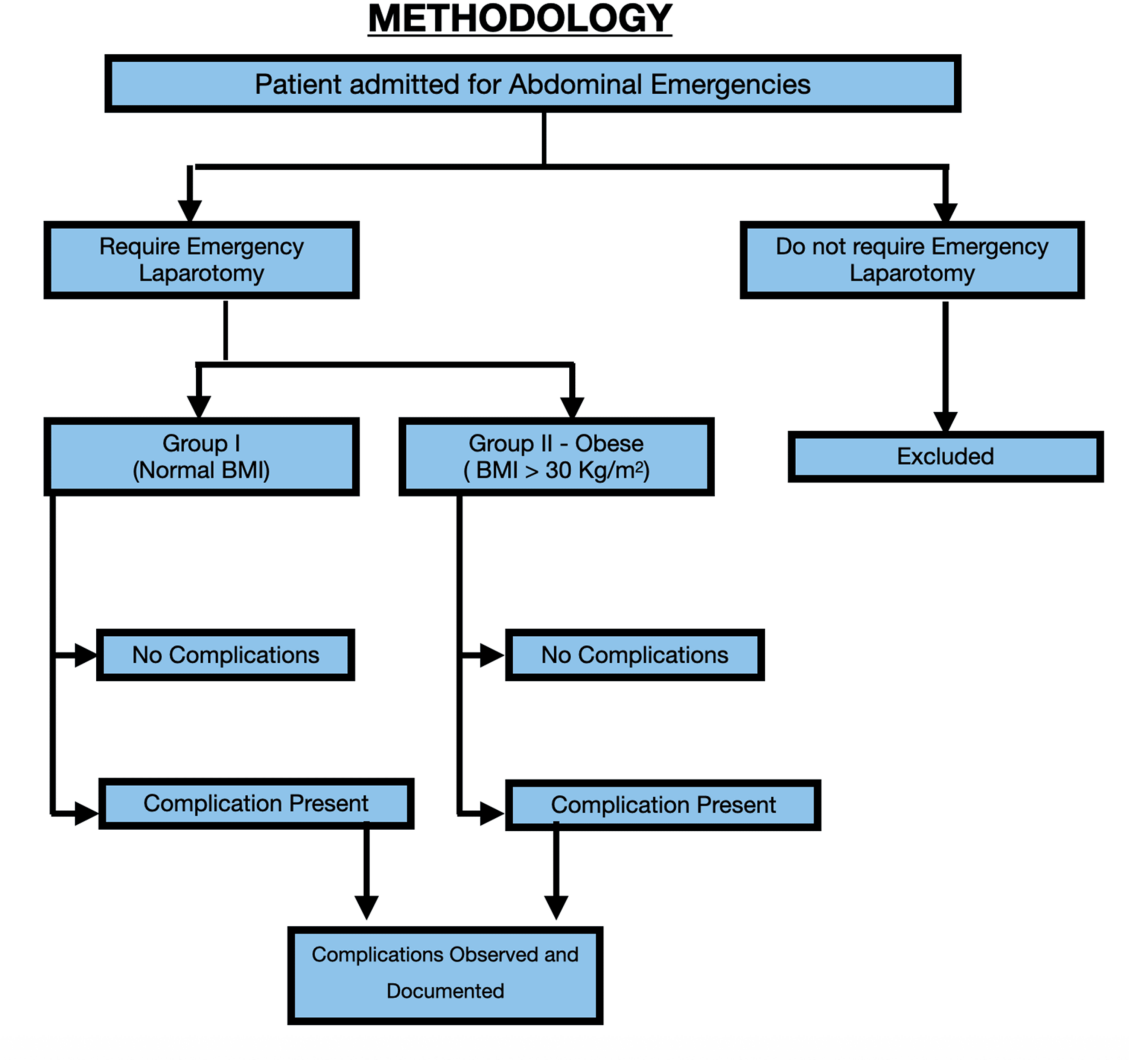
Methodology of the study.

This observational descriptive study was approved by the institutional ethics committee and conducted over 11 months from August 2022 to June 2023. A total of 72 emergency laparotomy patients were grouped by BMI, normal vs. obese, as per the WHO classification. Weight classification was assigned based upon BMI (kg/m^2^) and as defined by the World Health Organization (WHO), with BMI cutoff points for underweight (<18.5 kg/m^2^), normal weight (18.5-24.99 kg/m^2^), overweight (25-29.99 kg/m^2^), and obese (≥30 kg/m^2^) [13]. Comorbidities and surgical procedures were recorded, excluding laparoscopic cases and underweight patients (Figure 1). The Clavien-Dindo classification of surgical complications (CDC) was used to classify surgical complications [14]. Based on the CDC, the comprehensive complication index (CCI) was calculated for each patient to evaluate the true overall morbidity burden of a procedure [15].

## Results

Statistical analysis

Statistical Package for Social Sciences (SPSS) version 22.0 (Armonk, NY: IBM Corp.), released in 2013, for Windows, was used to perform statistical analyses.

Descriptive and inferential statistics

Descriptive analysis of all the explanatory and outcome parameters was done using frequency and proportions for categorical variables, whereas for continuous variables, the mean and SD was used. The Mann-Whitney U test was used to compare the mean age, surgery time, and length of hospital stay between normal BMI and obese subjects. Chi-square test was used to compare the gender distribution, comorbidity status, procedure performed, and post-operative complications between normal BMI and obese subjects. Multivariate logistic regression analysis was performed to predict the risk of post-operative complications among study subjects. The level of significance was set at p<0.05.

The study involved 72 participants, aged 18-88 years, with no statistically significant difference in the mean age between those with normal BMI (42.02±17.97) and the obese patients (46.3±16.43). Out of the total, 24 subjects were classified as obese, with a predominant female representation. The distribution of comorbidities showed a significantly higher distribution of T2 diabetes mellitus and hypertension among obese patients (Table 1).

**Table 1:** Distribution of comorbidity status between normal BMI and obese group using chi-square test. *Statistically significant values. Comparison done using chi-square test. T2DM: type 2 diabetes mellitus; IHD: ischemic heart disease; CKD: chronic kidney disease

Variables	Category	Normal BMI	Obese, n (%)	p-Value
Comorbidity, n (%)	T2DM	7 (14.60)	14 (58.30)	<0.001*
	HTN	12 (25.00)	12 (50.00)	0.03*
	IHD	0 (0.00)	1 (4.20)	0.15
	CKD	0 (0.00)	1 (4.20)	0.15
	Nil	32 (66.70)	7 (29.20)	0.003*

Table 2 shows a highly significant (p<0.001) difference in the distribution of post-operative complications according to the Clavien-Dindo classification, with a greater percentage seen among obese patients who developed grade 2 (41.7%) and grade 4 (37.5%) complications.

**Table 2:** Distribution of post-operative complications between normal BMI and obese groups using chi-square test. *Statistically significant values. Comparison done using chi-square test.

Variables	Category	Normal BMI, n (%)	Obese, n (%)	p-Value
Complications	Grade 2	2 (4.20)	10 (41.70)	<0.001*
	Grade 3	0 (0.00)	1 (4.20)	
	Grade 3a	1 (2.10)	2 (8.30)	
	Grade 4	4 (8.30)	9 (37.50)	
	Nil	41 (85.40)	2 (8.30)	

Table 3* *shows that the mean surgery time in normal BMI patients was 139.94 minutes, whereas in patients who were obese, it was 184.17 minutes, which showed high statistical significance (p<0.001) in this study.

**Table 3:** Comparison of the mean surgery time (in minutes) between the normal and obese group using Mann-Whitney U test. *Statistically significant value. Comparison done using Mann-Whitney U test.

Parameter	Groups	n	Mean	SD	Mean difference	p-Value
Surgery time	Normal BMI	48	139.94	44.17	-44.23	0.001*
	Obese	24	184.17	53.97		

Table 4 shows that the overall mean length of hospital stay among the normal BMI patients was 10.08 days, whereas the patients who were obese had a mean length of hospital stay of 14.00 days, which was highly statistically significant (p<0.001).

**Table 4:** Comparison of the length of hospital stay (in days) between normal BMI and obese group using Mann-Whitney U test. *Statistically significant value. Comparison done using Mann-Whitney U test.

Parameter	Groups	n	Mean	SD	Mean difference	p-Value
Hospital stay	Normal BMI	48	10.08	1.66	-3.92	<0.001*
	Obese	24	14	3.18		

Table 5 shows the association of specific surgical procedures and the development of post-operative complications, which were more frequent in distribution among the obese patients with a BMI >30 kg/m^2^. Nearly half (47.3%) of the obese patients who underwent surgical repair of small bowel perforation developed post-operative complications (p=0.04). More than half (52.7%) of the obese patients who had undergone resection and anastomosis with no stoma creation for intestinal obstruction had developed post-operative complications (p=0.03).

**Table 5:** Association of specific surgical procedures and the development of post-operative complications among the two study groups using chi-square test. Association was studied using chi-square test. Surgeries performed were as follows: S×1 - repair of small bowel perforation, S×2 - appendectomy with peritoneal lavage, S×3 - resection and anastomosis with no stoma, S×4 - hernial mesh repair for obstructed umbilical hernia, S×5 - repair of obstructed umbilical hernia, and S×6 - resection and anastomosis of ileum and ileostomy.

Surgery category	Normal BMI, n (%)	Obese, n (%)	p-Value
Sx1	3 (42.90)	6 (27.30)	0.44
Sx2	0 (0.00)	1 (4.50)	0.57
Sx3	3 (42.90)	5 (22.70)	0.3
Sx4	0 (0.00)	2 (9.10)	0.41
Sx5	0 (0.00)	4 (18.20)	0.22
Sx6	1 (14.30)	4 (18.20)	0.81

Table 6 compares the mean duration of the surgery in minutes for a particular surgery in the normal and obese groups. For the repair of small bowel perforation, appendectomy with peritoneal lavage, resection, and anastomosis with no stoma, the mean duration of surgery was significantly longer in obese subjects compared to normal BMI subjects, the p-values are <0.001, 0.01, and 0.04, respectively, indicating statistically significant differences. The obese group had notably longer durations compared to normal BMI subjects. However, there was no statistically significant difference in mean duration between the two groups with the other surgical procedures of hernial mesh repair for obstructed umbilical hernia, repair of obstructed umbilical hernia, and resection and anastomosis of ileum and ileostomy.

**Table 6:** Comparison of the mean duration of surgery (in minutes) for a particular surgery in the normal BMI and obese subjects using Mann-Whitney U test. *Statistically significant values. Comparison done using Mann-Whitney U test.

Surgery	Groups	n	Mean	SD	Mean difference	p-Value
Repair of small bowel perforation	Normal BMI	17	131.47	24.35	-58.53	<0.001*
	Obese	6	190	20.98		
Appendectomy with peritoneal lavage	Normal BMI	9	139.78	38.05	-87.22	0.01*
	Obese	1	240	.		
Resection and anastomosis with no stoma	Normal BMI	13	162.46	59.07	-73.54	0.04*
	Obese	5	236	70.92		
Hernial mesh repair for obstructed umbilical hernia	Normal BMI	2	130	42.43	-23.33	0.64
	Obese	3	153.33	51.32		
Repair of obstructed umbilical hernia	Normal BMI	1	140	.	-12.5	0.75
	Obese	4	152.5	32.02		
Resection and anastomosis of ileum and ileostomy	Normal BMI	6	118.67	57.02	-39.33	0.25
	Obese	5	158	48.17		

Table 7* *shows that higher BMI was a statistically significant and very strong predictor of post-operative complications. Subjects with higher BMI had over 100 times greater odds of developing complications compared to those with lower BMI, and this association was highly significant (p<0.001). Type 2 diabetes mellitus and hypertension were not statistically significant predictors in this study.

**Table 7:** Multivariate regression analysis to predict the risk of post-operative complications among study subjects. *Statistically significant values. T2DM: type 2 diabetes mellitus

Dependent variable	Independent variables	OR	95% CI for OR		p-Value
			Lower	Upper	
Complications	BMI	102.18	10.89	958.66	<0.001*
	T2DM	4.69	0.25	89.56	0.31
	HTN	16.19	0.66	394.33	0.09
	No comorbidity	53.74	1.12	2587.29	0.04*

## Discussion

This study investigated the effect of obesity on post-operative outcomes following emergency laparotomy. The results indicate that obese patients are at a higher risk of developing post-operative complications compared to their counterparts with normal BMI. Specifically, the study revealed that 41% of obese patients developed grade 2 post-operative complications according to the Clavien-Dindo classification, compared to only 4.2% of patients with normal BMI. This stark contrast highlights the increased vulnerability of obese individuals to adverse surgical outcomes. The Clavien-Dindo classification provides a standardized approach to grading the severity of post-operative complications. The higher incidence of complications in obese patients across various grades suggests that obesity not only increases the likelihood of complications but also their severity. The association between obesity and post-operative complications has been well-documented in the literature [16]. For instance, a study by Dindo et al. also used the Clavien-Dindo classification and found that obese patients undergoing major surgery were more likely to experience higher-grade complications [9]. Our findings corroborate these results, reinforcing that obesity is a significant risk factor in surgical settings.

The study also found a significant difference in the mean operative time between the two groups. The mean surgery time for obese patients was 184.17 minutes, compared to 139.94 minutes for patients with normal BMI. The prolonged operative time in obese patients is likely attributable to several factors. Excess adipose tissue can obscure anatomical landmarks, making surgical access more challenging and time-consuming. Additionally, the increased tissue bulk necessitates more extensive dissection and suturing, which further contributes to longer operative times. An increase in the operative time for the obese has also been reported in other studies [17-22]. Prolonged operative time is associated with several adverse outcomes, including an increased risk of infection, greater intra-operative blood loss, and higher post-operative morbidity. A study by Mullen et al. found that increased operative time is an independent risk factor for surgical site infections (SSIs) [23]. The current study found that obese patients had a mean hospital stay of 14.00 days, compared to 10.08 days for patients with normal BMI. The prolonged hospital stay in obese patients can be attributed to several factors, including the higher incidence of post-operative complications, delayed wound healing, and the need for more intensive post-operative care. The literature supports the association between obesity and extended hospital stays [24,25]. For example, a study by Tjeertes et al. found that obese patients undergoing surgery had longer hospital stays and were more likely to require intensive care unit (ICU) admission [17,26].

The study also examined the association between the type of surgical procedure and the development of post-operative complications. The 47.3% complication rate in obese patients after small bowel perforation repair and the risks associated with resection/anastomosis without stoma are consistent with broader evidence linking obesity to higher post-operative morbidity in gastrointestinal surgery. Kassahun et al.’s multivariate regression analysis of the factors identified to be significantly different in obese patients found that the odds ratio (OR) for anastomotic leakage was 5.5 [12]. This finding is particularly concerning, as it suggests that certain types of surgeries may pose a higher risk for obese patients. Performing any surgical procedure in obese patients in emergencies is technically challenging, and thus, the risk for the development of various complications is high [7,27]. For general surgery procedures, Yanquez et al. found an increased risk of complications in patients with obesity [28].

Clinical implications and recommendations

The findings from this study have several important clinical implications. First and foremost, they highlight the need for heightened awareness and proactive management of obesity as a significant risk factor in surgical patients. Given the increased morbidity and mortality associated with obesity, healthcare providers must implement targeted strategies to optimize the pre-operative, intra-operative, and post-operative care of obese patients.

Pre-operatively, efforts should be made to optimize the patient's medical condition, including the management of comorbidities such as T2DM and hypertension. Nutritional counseling and weight management programs should be offered to obese patients, particularly those undergoing elective surgery. Intra-operatively, the use of advanced surgical techniques, such as minimally invasive surgery, may help reduce operative times and the associated risks. Post-operatively, close monitoring for complications, early mobilization, and the use of evidence-based protocols, such as Enhanced Recovery After Surgery (ERAS), can help improve outcomes.

Limitations of the study

This study provides valuable insights into how obesity affects surgical outcomes, though it has some limitations. While the sample size is adequate for detecting statistical differences between groups, it remains relatively small, potentially limiting the broader applicability of the findings. Additionally, since the research was conducted at a single center, the patient population may not fully represent the general population. To strengthen these conclusions, future studies should include larger, more diverse participant groups and multi-center designs.

Furthermore, the study focuses exclusively on patients undergoing emergency laparotomy, a high-risk procedure; therefore, its findings may not apply to elective or minimally invasive surgeries. Future research should examine how obesity influences a wider range of surgical procedures, helping to identify specific factors that could reduce risks in different surgical scenarios.

Future research directions

Several areas warrant further investigation based on these findings. One key focus should be developing and evaluating interventions aimed at minimizing surgical risks for obese patients. Research into pre-operative weight loss programs, peri-operative glycemic control strategies, and the application of advanced surgical techniques could help mitigate complications. Additionally, more studies are needed to explore the biological mechanisms behind increased surgical risks in obese individuals. Understanding how inflammation, immune dysfunction, and impaired wound healing contribute to poor outcomes could pave the way for targeted therapeutic strategies, improving surgical success rates for this population.

Finally, the economic impact of obesity on healthcare, particularly in the context of surgery, is an important area for future research. Studies that quantify the additional costs associated with treating obese patients, including longer hospital stays, increased use of resources, insurance-related implications, and higher rates of readmission, could provide valuable information for healthcare policymakers and inform the development of cost-effective interventions.

## Conclusions

In conclusion, this study demonstrates that obesity significantly impacts post-operative outcomes in patients undergoing emergency laparotomy. Obese patients face a higher risk of complications, longer operative times, and extended hospital stays compared to patients with normal BMI. The presence of comorbidities such as T2DM and hypertension further exacerbates these risks, highlighting the need for comprehensive pre-operative assessment and optimization.
